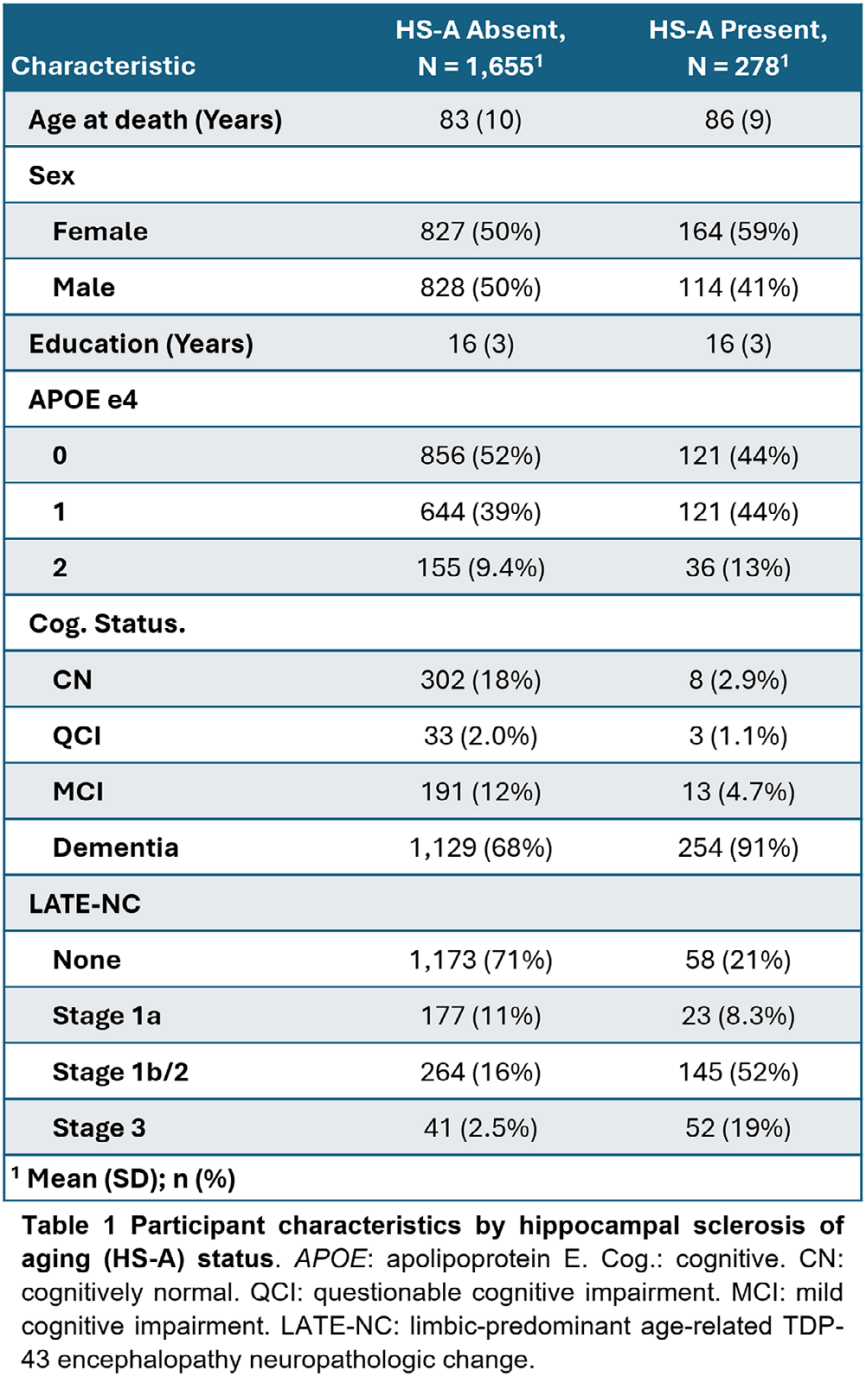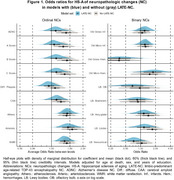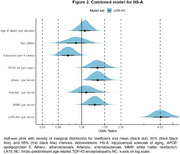# Examining associations of common neuropathologic changes with hippocampal sclerosis of aging

**DOI:** 10.1002/alz70855_097024

**Published:** 2025-12-23

**Authors:** Davis C. Woodworth, Jerry Jierui Lou, William H Yong, Elizabeth Head, María M. M. Corrada, Peter T Nelson, Seyed Ahmad Sajjadi

**Affiliations:** ^1^ University of California, Irvine, Irvine, CA, USA; ^2^ University of Kentucky, Lexington, KY, USA

## Abstract

**Background:**

Hippocampal sclerosis of aging (HS‐A) is a neuropathologic change (NC) of neuronal dropout and gliosis in the hippocampus affecting up to 20% of elderly persons with dementia. While HS‐A has been found to be strongly associated with limbic‐predominant age‐related TDP‐43 encephalopathy (LATE‐NC), associations with other NCs have been inconsistent across previous studies. Additionally, because of how strongly LATE‐NC and HS‐A are related, it is important to account for LATE‐NC when examining the associations between other NCs and HS‐A. The goal of this study was to examine how other common NCs are associated with HS‐A, both before and after accounting for LATE‐NC.

**Method:**

We used data from participants in the National Alzheimer's Coordinating Center (NACC), excluding participants with frontotemporal lobar degeneration (FTLD). We examined associations of Alzheimer's disease (ADNC), Lewy bodies (LB), and cerebrovascular pathologies, with HS‐A, both with and without accounting for LATE‐NC stage. We used Bayesian multilevel logistic regression models with monotonic modeling for ordinal predictors and report odds ratios (OR) along with 95% credibility intervals (CI).

**Result:**

We used data from 1933 participants (average age at death of 83 years, Table 1). HS‐A was present in 278 participants (14.4%). As expected, LATE‐NC was strongly associated with HS‐A (average OR=3.7 across levels, 95% CI=[2.8,5.01]). Figure 1 shows ORs from separate models for each of the NC predictors. While ADNC showed a modest association with HS‐A in models without LATE‐NC (OR=1.39, CI=[1.10, 1.78]), this association was reduced when accounting for LATE‐NC (OR=1.11, CI=[0.85, 1.45]); we also found similar results for the ADNC‐related A/B/C scores and limbic LBs. However, the cerebrovascular NCs of atherosclerosis, arteriolosclerosis, and white matter rarefaction (WMR), were associated with HS‐A both without and with accounting for LATE‐NC (ORs∼1.5 per level). In a combined model including LATE‐NC and cerebrovascular NCs (Figure 2), LATE‐NC remained strongly related to HS‐A, but global cerebrovascular NCs, as well as *APOE*‐ε4 (increased odds), and education (decreased odds) were also associated with HS‐A.

**Conclusion:**

HS‐A is strongly, uniquely (compared to other NCs), and likely causally, related to LATE‐NC. The vascular NCs of arteriolosclerosis, atherosclerosis, and WMR, are also associated with HS‐A, even after accounting for LATE‐NC.